# Morphometric approach to 3D soft-tissue craniofacial analysis and classification of ethnicity, sex, and age

**DOI:** 10.1371/journal.pone.0228402

**Published:** 2020-04-09

**Authors:** Olalekan Agbolade, Azree Nazri, Razali Yaakob, Abdul Azim Ghani, Yoke Kqueen Cheah

**Affiliations:** 1 Department of Computer Science, Faculty of Computer Science & IT, Universiti Putra Malaysia, Seri Kembangan, Selangor, Malaysia; 2 Department of Software Engineering, Faculty of Computer Science & IT, Universiti Putra Malaysia, Seri Kembangan, Selangor, Malaysia; 3 Department of Biomedical Science, Faculty of Medicine and Health Sciences, Universiti Putra Malaysia, Seri Kembangan, Selangor, Malaysia; Liverpool John Moores University, UNITED KINGDOM

## Abstract

**Background:**

The application of three-dimensional scan models offers a useful resource for studying craniofacial variation. The complex mathematical analysis for facial point acquisition in three-dimensional models has made many craniofacial assessments laborious.

**Method:**

This study investigates three-dimensional (3D) soft-tissue craniofacial variation, with relation to ethnicity, sex and age variables in British and Irish white Europeans. This utilizes a geometric morphometric approach on a subsampled dataset comprising 292 scans, taken from a Liverpool-York Head Model database. Shape variation and analysis of each variable are tested using 20 anchor anatomical landmarks and 480 sliding semi-landmarks.

**Results:**

Significant ethnicity, sex, and age differences are observed for measurement covering major aspects of the craniofacial shape. The ethnicity shows subtle significant differences compared to sex and age; even though it presents the lowest classification accuracy. The magnitude of dimorphism in sex is revealed in the facial, nasal and crania measurement. Significant shape differences are also seen at each age group, with some distinct dimorphic features present in the age groups.

**Conclusions:**

The patterns of shape variation show that white British individuals have a more rounded head shape, whereas white Irish individuals have a narrower head shape. White British persons also demonstrate higher classification accuracy. Regarding sex patterns, males are relatively larger than females, especially in the mouth and nasal regions. Females presented with higher classification accuracy than males. The differences in the chin, mouth, nose, crania, and forehead emerge from different growth rates between the groups. Classification accuracy is best for children and senior adult age groups.

## Introduction

Morphometrics is the study of shape variation and its covariation with other variables [1,2]. According to Dean et al. [3], morphometrics was traditionally the application of multivariate statistical analyses to a set of quantitative variables such as length, width, height and, angle. But advances in morphometrics have shifted focus to the Cartesian coordinates of anatomical points that might be used to define more traditional measurements. Morphometrics examines shape variation, group differences in shape, the central tendency of shape, and associations of shape with extrinsic factors [4]. The latest approach to shape analyses is geometric morphometric. This is directly based on the digitized x,y,z-coordinate positions of landmarks, points representing the spatial positions of putatively homologous structures in two- or three-dimensions; whereas conventional morphometric studies utilize distances as variables [1,2,5].

A flexible and mathematically rigorous interpolation technique of D’Arcy Thompson’s transformation grids [6], called thin plate-spline (TPS), was brought into morphometrics. This ensures that the corresponding points of the starting and target form appear precisely in corresponding positions in relation to the transformed and untransformed grids [7]. With the application of iterative closest point (ICP) method, landmark correspondence can iteratively be registered in the vicinity of a landmark with a re-weighted error function. This computes the amount of deformation between two shape configurations as quantified by TPS function through the integral of the squared second derivatives of that deformation [8]. The use of three-dimensional head and face images in morphometrics does not only give room to cover a wider area of the human craniofacial region but also retains all the geometric information of the object descriptors [9,10].

Craniofacial measurement traditionally has reliance on simple distances and angles between anatomical landmarks. These give only a limited representation of the surface under study [11]. Advances in three-dimensional image analysis have now achieved rapid, automatic measurement of the entire outer surface of the craniofacial soft-tissue [12,13].

Characterizing human craniofacial shape for ethnicity classification, sex dimorphism, and age estimation is of interest to numerous fields, including forensics [14], anthropology [15,16], cognitive science [17,18], and orthodontics [19,20]. Study of adult face shape in population samples with mixed West African and European ancestry from three locations by [21] estimated ethnicity for only 9.6% of shape variation, while sex differences accounted for 12.9% of shape variation among individuals. This multifactorial craniofacial shape differences result from a combination of hormonal influences and inherent genetic factors [22]. An ethnicity classification was applied in public health by [23] to subdivide populations into groups of common origin. They presented a positive predicted value between 0.70 and 0.96, and a negative predicted value between 0.96 and 1. Ethnicity and gender identifications were investigated using multimodal Asian and Non-Asian faces by [24],exploring two modalities of human faces: intensity and range. The range provided an effective capability for the classification. Asides ancestry or ethnicity classification, many studies have been carried out involving sexual dimorphism in the craniofacial soft tissue of children [11,25], young adults [19,26,27] and adults [18,21,28]. The consensus from the studies is that male faces are larger and characterized by more prominent nasal, chin, and forehead regions.

However, there is a limitation of amalgamated data from mixed ages to create a single classification model or a single pair of prototypes [11]. Age estimation is usually approached as a classification or regression problem by prediction of age or age group from an image in computer vision [11]. Prediction of age and other traits from 3D shape was done by [29], by systematically adjusting the input image until its predicted age matched a target age. Also, simulated 3D facial ageing by caricaturing was carried out by [30,31].

However, since a big part of biological variability cannot be assessed by using only anatomical landmarks [32], in order to quantify complex shapes, sliding semi-landmarks were developed which could be placed on surfaces [33] or curves [1,33]. This approach generates landmarks that are spatially homologous after sliding [34], which can be optimized by minimizing the bending energy [35,36] or the Procrustes distance [37,38]. As the human face hosts features that act as a central interface for identification, more landmarks are needed to characterize biological shape variation [32]. Due to this, we have characterized biological shape variation for ethnicity, sex, and age using a total of 500 three dimensional landmarks, which incorporate sliding semi-landmarks to promote a computationally efficient workflow.

The aim of this study was to first extend the computational deformation process by [7]. Surface semi-landmarks were projected from the template object to the target object. As an alternative to the complex workflow demonstrated by [32], a simpler workflow was presented using Viewbox 4.0, which iteratively slid the semi-landmark to a relaxed point. Secondly, the landmark data acquired was further analysed to independently investigate the shape and size of variation between ethnicity, sex, and age groups using principal component analysis (PCA) for dimensionality reduction and features selection. Thirdly, anthropometric measurement was further performed using Euclidean Distance Matrix Analysis (EDMA) to measure the length of the line segment connecting selected anatomical points and localize the site of major variations in the sample groups. Fourthly, allometry was examined for each group separately to investigate effect of size unto shape. Lastly, the features selected were further used for classification using discriminant analysis.

## Materials and methods

For ethics approval, the use of human subjects was approved by the committee in charge of the Liverpool-York Head Model in Alder Hey Craniofacial Unit, Liverpool, UK. Therefore, there is no institutional review board approval required to use the public dataset, asides the user license agreement signed between the two parties. Regarding the use of subjects, we have contacted the head of data access committee of the dataset for more clarifications. He clarified that there is no restriction on the use of any or all the subjects under the CC-BY license and also that all subjects signed forms with consent to publish.

### Dataset and description

This study used a randomly selected sub-sample of 292 (white British = 234, white Irish = 58) craniofacial images from the Headspace dataset. Only white British and white Irish descent, all of whom are wearing tight-fitting latex caps [39] are sub-sampled. A 3dMD five-camera system was used to create a 3D triangular surface for each subject composed of typically 180K vertices and 360K triangles acquired at Alder Hey Hospital, Liverpool. The dataset metadata is comprised of ethnicity, sex, age, eye color, and any craniofacial surgery/trauma condition. This is the first public shape and texture 3D morphable model of the full human head called Liverpool-York Head Model (LYHM) [39]. These particular ethnicities’ demographics are chosen because of their morphological characteristics. The two populations selected also have the highest sample sizes which could be used to morphometrically characterise shape variation. Table 1 presents the full details of the demographic information of the sub-sample dataset. The age class in years is sub-divided into five categories: children (below 13 years), teenagers (13–19 years), young adults (20–29 years), adults (30–49 years), and senior adults (50 years and above).

**Table 1:** Demographic information of the sub-sample dataset.

	White British				White Irish					
	Male		Female		Male		Female		Total	
Age Group	N	%	N	%	N	%	N	%	N	%
<13	21	20.59	24	18.18	1	3.7	1	3.23	47	16.09
13–19	13	12.74	11	8.34	0	0	3	9.68	27	9.25
20–29	26	25.49	32	24.24	6	22.22	7	22.58	71	24.31
30–49	21	20.59	33	25	14	51.86	12	38.71	80	27.40
50>	21	20.59	32	24.24	6	22.22	8	25.80	67	22.95
Total	102	100	132	100	27	100	31	100	292	100

### Creating template mesh

**Fig 1:**
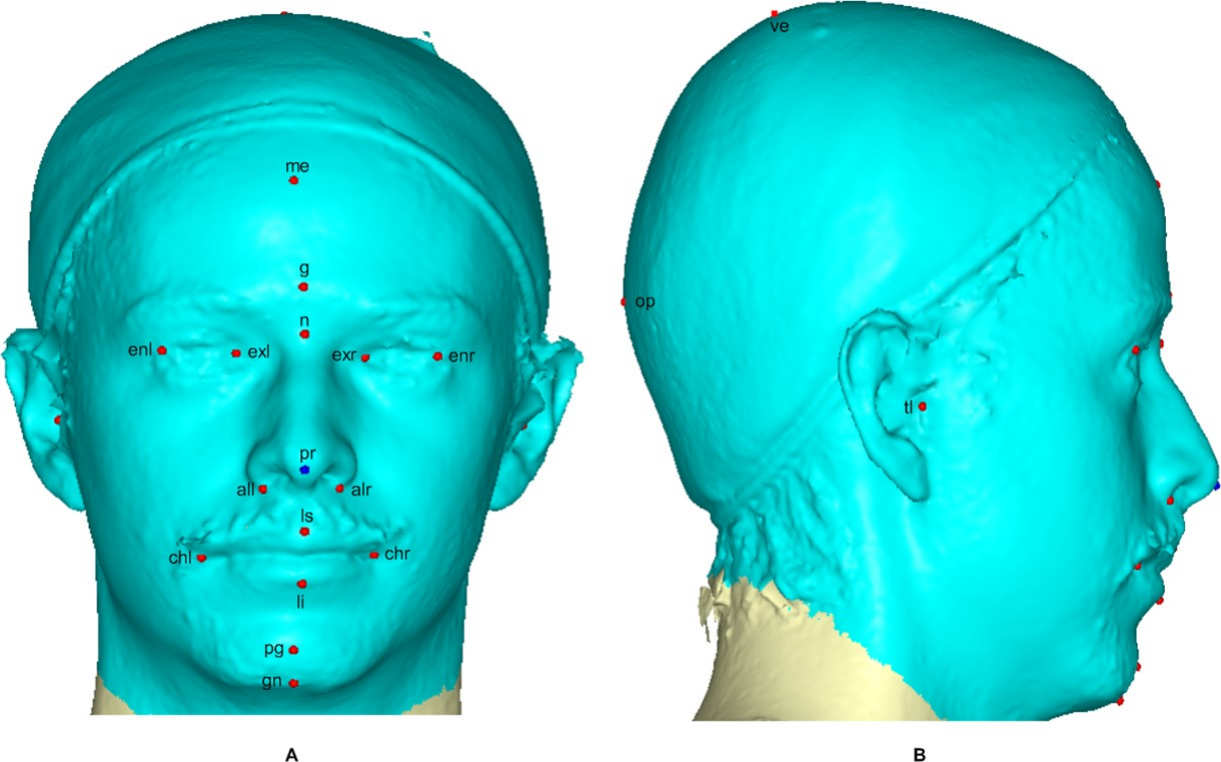
A three-dimensional mesh template of frontal, lateralregions. The 20 anchor anatomical landmarks are shown in red. The blue are on the pronasale indicates the point where the semi-landmarks begin the sliding process.

**Fig 2:**
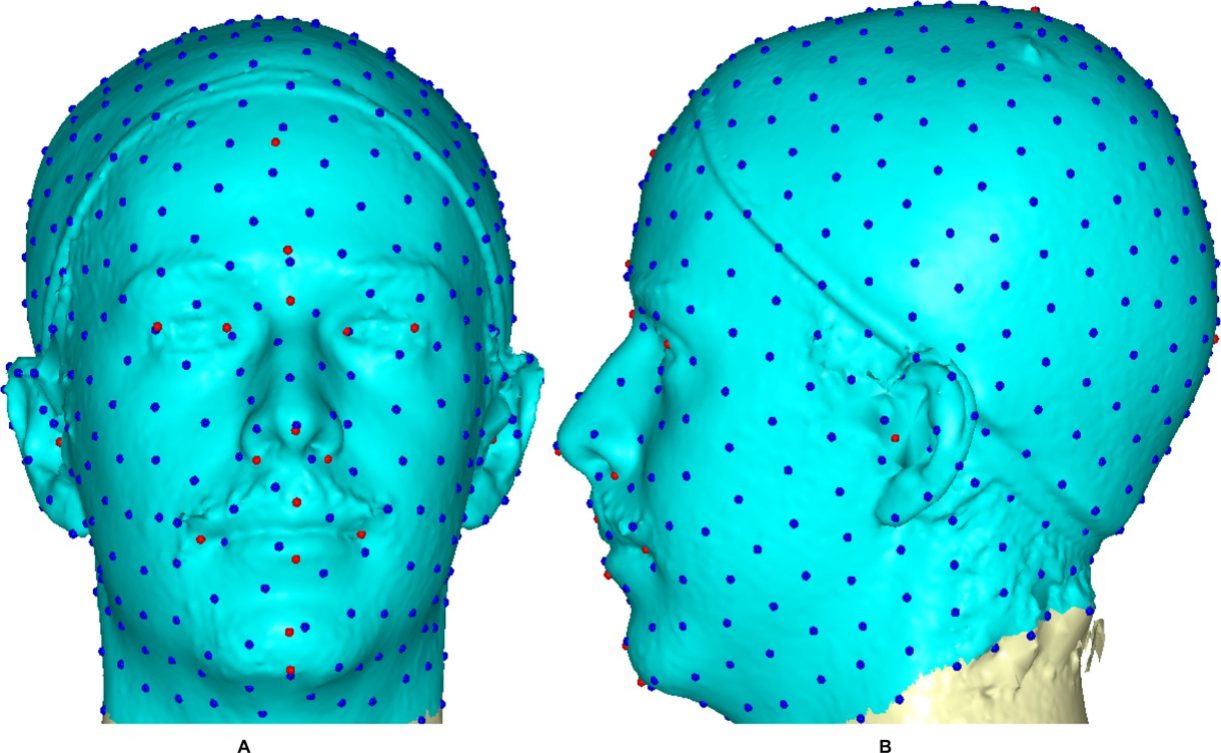
A three-dimensional mesh template of the reference model with 500 landmarks. Showing 20 anchor anatomical points (red color) and 480 semi-landmarks (blue color) with 1.5 mm radius: (A) Frontal view. (B) Lateral view.

A 3D mesh template was created by manually locating twenty anatomical points on a 3D head and face (Fig 1) according to facial landmark standards [40–42] (for more detail, see Table 2). The 20 anchor anatomical landmarks were not subjected to sliding but were used to establish the warping fields that would be used for minimizing the bending energy. Due to its easy of detection and pose correction [43] and its invariance to the facial expression of nose tip [44], the pronasale has been selected as the most robust and prominent landmark point. The sliding points begin to spread across the craniofacial surface from the nose tip. Using this anchor point (the pronasale), 480 semi-landmarks were automatically generated with the overlapping on the pronasale shown blue. These were first randomly placed on the craniofacial mesh before being uniformly distributed on the selected craniofacial surface using the locational positions of the anchor anatomical points with a 1.5mm radius to accommodate all the 500 points (see S1 Data) (Fig 2). To quantify the morphological for the complex, three-dimensional traits of both reference and target shapes, we used geometric morphometric tools based on previously reported landmark-based methodologies in [45–51] and the method was fully implemented in Viewbox 4.0 [49].

**Table 2:** Anchor anatomical points and descriptions.

No	Anchor Landmarks	Notation	Description
1	Endocanthion left	enl	Left most medial point of the palpebral fissure, at the inner commissure of the eye.
2	Exocanthion left	exl	Left most lateral point of the palpebral fissure, at the outer commissure of the eye.
3	Exocanthion right	exr	Right most lateral point of the palpebral fissure, at the outer commissure of the eye.
4	Endocanthion right	enr	Right most medial point of the palpebral fissure, at the inner commissure of the eye.
5	Metopion	me	Median point, instrumentally determined on the frontal head as the greatest elevation from a cord between nasion and glabella.
6	Glabella	g	The most prominent midline point of the forehead between the brow ridges
7	Nasion	n	The point in the midline of the nasal radix and nasofrontal
8	Pronasale	pr	The most prominent point on the nasal tip
9	Alare left	all	Left most lateral point on the nasal ala.
10	Alare right	alr	Right most lateral point on the nasal ala.
11	Cheilion left	chl	Left outer corners of the mouth where the outer edges of the upper and lower vermilions meet.
12	Cheilion right	chr	Right outer corners of the mouth where the outer edges of the upper and lower vermilions meet.
13	Labiale superius	ls	Midpoint of the vermilion border of the upper lip.
14	Labiale inferius	li	Midpoint of the vermilion border of the lower lip.
15	Pogonion	pg	The most prominent midline point of the soft tissue chin pad
16	Gnathion	gn	The most anterior inferior midline point on the soft tissue chin contour
17	Tragion left	tl	The left notch in the superior margin of each tragus
18	Tragion right	tr	The right notch in the superior margin of each tragus
19	Opisthocranion	op	Most posterior median point of the occipital bone, instrumentally determined as the greatest chord length from glabella
20	Vertex	ve	Most superior point of the head.

### Multi-point warping

The geometry of curves and surfaces is easier in 2D or 3D but it is less easy to define semi-landmarks for non-planar surfaces in 3D [52], as they are not guaranteed to be homologous after first placement. However, this could be achieved by subjecting the semi-landmarks to sliding in the direction that reduces shape variance, thus closely positioning the points at the same locations in the 3D space. The sliding step is important, as it places the landmarks in positions where they correspond better to each other between individuals [37]. These semi-landmarks were allowed to slide on the curves and the surface mesh of each target using TPS warping of the template, which positioned the reference points on the target craniofacial mesh by minimizing the bending energy.

According to Bookstein [7], physical steel takes a bending form with a small displacement. This is because the function (*x*,*y*,*z*) is the configuration of the lowest physical bending energy which is consistent with the given constraints. In this 3D head and face deformation, the transformation of TPS was done mathematically by interpolation of smooth mapping of *h* from $R^{3}\to R^{3}$. This is a selected set of corresponding points {P_*Ri*_,P_*Ti*_}, *i* = 1,…,*N* on the faces of the reference object and target that minimizes the bending energy function E(*h*) using the following interpolation conditions [1,7,53]:

$$\begin{array}{l} E(h)={∭}_{{\mathbb{R}}^{3}}{\left((\frac{\partial^{2}h}{\partial x^{2}}\right)}^{2}+{\left(\frac{\partial^{2}h}{\partial y^{2}}\right)}^{2}+{\left(\frac{\partial^{2}h}{\partial z^{2}}\right)}^{2}+\\ 2{\left(\frac{\partial^{2}h}{\partial xy}\right)}^{2}+2{\left(\frac{\partial^{2}h}{\partial xz}\right)}^{2}+2{\left(\frac{\partial^{2}h}{\partial yz}\right)}^{2})dxdydz,\\ s.t.\;h\left(P_{Ti}\right)=P_{Ri},i=1,\ldots ,M \end{array}$$ (1)

where P_*Ti*_ is the target object, P_*Ri*_ is the reference object for the sets of corresponding points, and *h* is the bending energy function that minimizes the non-negative quantity of the interpolation of the integral bending norm or the integral quadratic variation E(*h*). The TPS method now decomposes each component into affine and non-affine components, such that

$$h(P_{h})=\Psi (P_{h})K+P_{h}\Gamma$$ (2)

where P_*h*_ are the homogeneous coordinate points on the target 3D surface, and Ψ(P_*h*_) = (Ψ_1_(P_*h*_),Ψ_2_(P_*h*_),…,Ψ_*M*_(P_*h*_)) is a 1 × M kernel vector of TPS of the form:

$$\Psi_{w}(P_{h})=∥P_{h}-P_{Tw}∥$$ (3)

K is a M × 4 non-affine warping coefficient matrix, and Γ is a homogeneous affine transformation of a 4x4 matrix. The energy function is minimized to find the optimum solution to Eq 4, if the interpolation condition in Eq 1 is not met.

$$E(\beta ,K,\Psi )=\frac{1}{M}\sum_{J=1}^{M}\|h(P_{Tj})-P_{Rj}\|+\beta E(h)$$ (4)

The interpolation conditions in Eq 1 are satisfied if the smoothing regularization term *β* is zero; Γ and K are TPS parameters obtained by solving the linear equation:

$$\left(\begin{array}{cc} \Psi & P_{R}\\ P_{R}^{T} & 0 \end{array}\right)\left(\begin{array}{c} K\\ \Gamma \end{array}\right)=\left(\begin{array}{c} P_{T}\\ 0 \end{array}\right)$$ (5)

**Fig 3:**
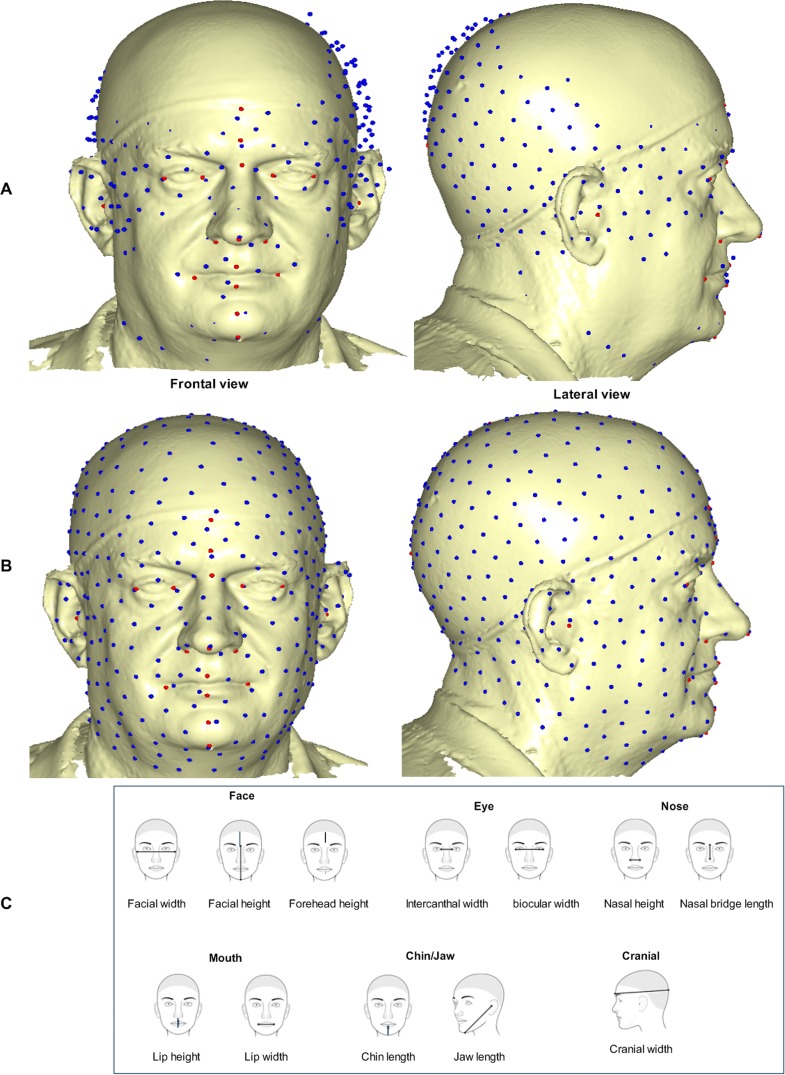
Sliding semi-landmark warped on target facial surface and distance measurement. (A) Partial sliding on target mesh–first iteration. (B) Complete and homologous warping on target mesh–sixth iteration. (C) Approximate location of selected distance measurement for six regions redacted from [41]. Showing face (facial width, facial height, and forehead height); eye (intercanthal width and biocular width); nose (nasal height and nasal bridge length); mouth (lip height and lip width); chin/jaw (chin height and jaw length); and cranial (cranial length).

Ψ is a M×M matrix with components Ψ_*wl*_ = ‖P_*Tw*_−P_*Tl*_‖ *and* P_*R*_ is a M×4 matrix in which each row is the homogeneous coordinate of the point P_*Ri*_, *i* = 1,…,*M*. Using Eq 2, the target craniofacial mesh P_*Ti*_ is deformed to the reference mesh P_*Ri*_. The bending energy was applied, and the process was iterated for six cycles to have optimum sliding of the points on the craniofacial surface which gives points relaxed. This changed the bending energy from the initial value *E*_*i*_ to the final value *E*_*f*_ after six complete iterations. This means that the semi-landmarks can be treated in the same way as homologous landmarks in downstream analyses. Since warping may result in points that do not lie directly on the craniofacial surface on the target mesh, the transferred points were projected onto the closest point on the mesh surface. This was done using the ICP method [43], which aims to iteratively minimize the mean square error between two point sets. If the distance between the two points is within the acceptable threshold, then the closest point is determined as the corresponding point [54]. The homologous landmark warping *H*_*K*Γ_ after six complete iterations is, therefore:

$$H_{K\Gamma }=E_{f-i}\left(\begin{array}{c} K\\ \Gamma \end{array}\right)$$ (6)

where

$$\left(\begin{array}{c} K\\ \Gamma \end{array}\right)={\left(\begin{array}{cc} \Psi & P_{R}\\ P_{R}^{T} & 0 \end{array}\right)}^{-1}\left(\begin{array}{c} P_{T}\\ 0 \end{array}\right),$$ (7)

is the linear TPS equation obtained during the surface deformation of the target mesh to the reference mesh before convergence was finally reached and *E*_*f−i*_ = *E*_*f*_−*E*_*i*_ of six complete iterations. The first iteration showed a partial distribution of sliding points on the target surface mesh (Fig 3A). This was automatically repeated until the optimum homologous result was achieved using an exponential decay sliding step of a hundred to five percent. During the relaxation of the spline, the semi-landmarks were slid along the surface and the curve tangent structures, rather than on the surfaces or the curves which reduced the computational effort. This makes the minimization problem become linear, since sliding along the tangents let the semi-landmarks slip off the data [33]. The target surface mesh is now treated as a set of homologous points (Fig 3B). Note that we did not construct a new deformable mathematical equation from scratch but simply extended the standard deformable method that has been established by [1].

The steps in this algorithm can be summarised as follows:

1. Anatomical fixed points (20) were identified and digitized on the template craniofacial mesh and a prominent point (the pronasale) was identified.
2. Semi-landmarks (480) were automatically generated and placed along the curves located at a uniform distance along each curve for sliding in step (5).
3. These semi-landmarks were first randomly placed and then uniformly distributed on the selected reference surface mesh, starting from the selected prominent point.
4. The reference facial model was warped to each target mesh configuration using a TPS transformation, and the surface semi-landmark was projected from the reference facial mesh to the target facial mesh.
5. The surface and curve semi-landmarks were then slid together in the direction that minimized the bending energy between each target configuration and the reference object. This was done iteratively in six complete cycles, in order to ensure convergence and optimum smoothness. This gave a homologous representation of the reference mesh.
6. A Generalized Procrustes analysis (GPA) of the landmark data was performed and an error assessment was computed using a Procrustes ANOVA in MorphoJ.

### PCA and distance analysis

Principal Components Analysis (PCA) is used for dimensionality reduction. The total principal components (PCs) computed during the reduction process is 300PCs. Among these, only 180PCs which have been observed to have the highest ranking eigenvectors are selected for further analyses. This is based on the eigenvalues from random data of the principal components (see S2 Data).

Shape differences among the groups are studied using the aligned coordinates to perform a PCA to describe major trends in shape; between white British and white Irish, between males and females, and among the age classes. The PCs obtained from these variables are known as relative warps [5]. The difference from mean shape as expressed by variation along the relative warp axes are the intuitive deformation grid diagrams [55]. To visualise ethnicity, sex, and age differences; graphical representations of shape differences are generated as deformation grids of the mean group shape relative to the reference configuration (i.e. consensus configuration). To visualise how shape changes among the groups, we plot lollipop graphs which show the shifts of landmark positions with straight lines. The length and direction of the line indicate the movement of the respective landmark in the mean shape.

Due to the visual interpretation of lollipop graph, we further employed the method of Euclidean Distance Matrix Analysis (EDMA) [56,57] to measure the length of the line segment connecting the selected anatomical points (Fig 3C) [41] using PAST version 2.17 (see S3 Data) and we take the log of all distances. EDMA first calculates all the possible Euclidean distances between the selected anchor landmarks, computed on the mean shape of each group. It does not only provide an objective measurement of shape differences but also localizes the sites of major variations by suggesting which of the landmarks are more involved in the form difference [58].

### Error assessment

The process of landmark coordinate extraction is always associated with some degree of measurement error. This can be as a result of non-coplanarity of landmarks, inconsistent of specimens relative to the plane of digitization, or difficulty in pinpointing the landmark locus [59]. Landmark measurement error can be minimized by careful landmark selection, but can never be totally eliminated. In assessing measurement error, thirty randomly selected images from both ethnicities were taken and digitized each image twice by the same operator following the method in [60–62]. These were done for both manual and sliding semi-landmarks, followed by Procrustes superimposition on the landmark data using three partitions: fixed anatomical landmarks, sliding semi-landmarks, and overall landmarks; after the specified effects have been included [60]. Procrustes ANOVA was assessed to quantify the relative amounts of variation of shape and measurement error among soft-tissue craniofacial and population [60]. Although several other error measurement methods were suggested by Fruciano [63], the measurement error for this study was assessed using a Procrustes ANOVA. This technique [60,64] was implemented in morphometrics to analyze measurement errors [65–67] using MorphoJ, which was achieved through the minimization of the squared sum of the distances of all objects and the consensus configuration [63]. It is crucial that the factors are accurately specified because of their hierarchical model, and therefore the order of the effects (first ethnicity, second sex, third age, followed by the individual) is important.

### DA, CVA and allometry

Analyses for discrimination and allometric patterns are conducted on data averaged for ethnicity, sex, and age. Canonical variate analysis (CVA) and discriminant analysis (DA) are used to test group differences, to plot their differences, and to predict group classification. Using MorphoJ, CVA was performed to test group differences. Furthermore, using PAST, DA was performed by computing cross-validated classification tables to find a set of axes that grants the greatest ability possible to discriminate between two or more groups [68]. Its main purpose is to achieve a predictive classification of each group by estimating the discriminant functions that best discriminate between groups, and computing their classification scores. The accuracy of the classifications was finally evaluated using a cross-validation analysis. The significance of discriminant functions is tested using Wilks’ lambda and F value.

Using Procrustes distance with 10,000 permutations, we assess the statistical significance of the pairwise difference in mean shape; this comes along with Mahalanobis distance but not considered in this study. Because there may be an interaction between the size and shape in craniofacial morphology due to changes in shape associated with size differences [64], we assessed allometry. Through allometry, we tested the statistical significant proportion of morphological variation in the symmetric components using a multivariate regression of shape onto centroid size. Due to the fact that their allometric trajectories could have group-specific slopes or intercepts, we examined allometry for each group separately. Subsequently, by computing non-parametric multivariate analysis of variance (MANOVA), differences in effects and size are examined and the effect of size on shape was corrected. In addition, they are processed by means of principal components analysis to reduce the number of variables. Out of the entire set of principal components, subsets were selected that account for 99.98% of the total variance.

A full MANOVA with ethnicity as groups, the size as covariate and the ethnicity-by-size interaction term included was performed. We further applied the same on sex and age using the size as covariate to test their interaction. The allometric trajectories are parallel if there is no significance [69], which indicates a similar pattern in the allometry across groups [45]. Lastly, the MANOVA was recomputed after removing the nonsignificant interaction term (ethnicity × size), and the ethnicity effect tested differences in regression intercepts. This is useful for the verification of whether differences in shape are the result of size variation only [69]. The aim is to test each group after removing the variance in shape accounted for by the covariate size. In so doing, we may verify if the differences in shape are actually as a result of size variation only. This increases statistical power and makes explanatory simpler models by controlling one factor while testing for another. Furthermore, a pooled within-group regression was performed in MorphoJ, resulting in a sample of ‘size corrected’ shapes according to group-specific parallel allometric trajectories [70].

## Experiment results

After the step-by-step methods in facial surface deformation of semi-landmarks in Viewbox 4.0, the analysis, visualisation and classification of the experiment are performed using MorphoJ 1.06d [71], and PAST 3.0 [72].

### PCA results

**Fig 4:**
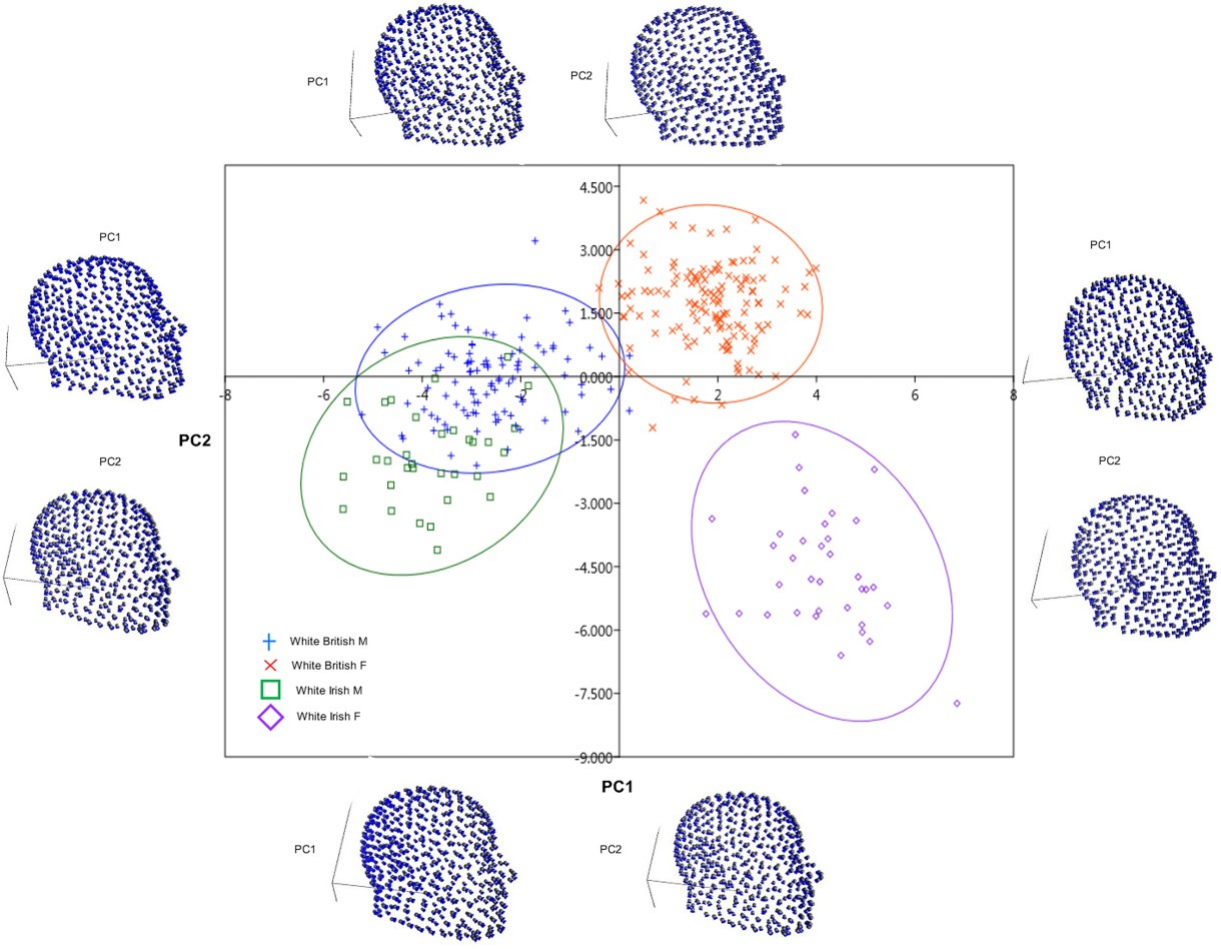
Shape variation (PC1 vs PC2) and corresponding relative warps. Centre: Showing the first two principal components of the shape variation. The ellipses represent 95% confidence intervals. Sides: Craniofacial deformation representing variation along first two PCs (Upper: white British males, Lower: white British females, Right: white Irish males, Left: white Irish females).

From the scatter plot of PC1 and PC2 scores (Fig 4), white British females and white Irish females separate along the positive axis of PC1 and PC2; whereas white British males and white Irish males separate along the negative axes of PC1 and PC2. The white British take the upper dimension while the white Irish take the lower dimension. Indeed, from the study of PCA, white British males are sparingly separated from white Irish males with some overlapping. This is as a result of the small sample size in Irish samples. Generally, PC1 accounted for 43.68% while PC2 accounted for 13.94% of the total variance. The relative warp deformations showed shape variation along PC1 and PC2. The PC1 and PC2 of white British males accounted for 54.29% and 11.24%, respectively; PC1 and PC2 of white British females accounted for 35.56% and 16.21%, respectively; PC1 and PC2 of white Irish males accounted for 32.74% and 17.07%, respectively; and PC1 and PC2 of white Irish females accounted for 31.26% and 18.26%, respectively.

### Procrustes ANOVA

The results for shape are reported in Table 3 and the effects are significant (all with p < 0.0001). For manual landmarks, the main effect of ethnicity is statistically significant and explains only 4.71% of the total variance. In addition, differences in craniofacial shape between sex, age, and among individuals are significant; and explained 1.30%, 8.04%, and 85.51%, respectively. The digitizing error accounted for 0.45% of the total variance. For sliding semi-landmarks, the main effect of ethnicity is statistically significant and explained only 3.07% of the total variance. In addition, differences in craniofacial shape between sex, age, and among individuals are significant; and explained 1.75%, 8.72%, and 86.13%, respectively. The digitizing error accounted for 0.33% of the total variance. For overall landmarks, the main effect of ethnicity is statistically significant and explained only 3.12% of the total variance. In addition, differences in craniofacial shape between sex, age, and among individuals are significant; and explained 1.72%, 8.69%, and 86.13%, respectively. The digitizing error accounted for 0.33% of the total variance.

**Table 3:** Procrustes ANOVAs for craniofacial shape with digitization error.

Effect	Var (%)	SS	MS	DF	F	P
**Manual Landmarks**						
Ethnicity	4.71	0.1195676	0.002255993	53	15.75	< .0001
Sex	1.30	0.03300793	0.000622791	53	4.35	< .0001
Age	8.04	0.20405101	0.000962505	212	6.72	< .0001
Individual	85.51	2.17086953	0.000143216	15158	3.05	< .0001
Digitizing Error	0.45	0.01135599	0.000053566	212		
Total	100	2.53885206	0.004038071	15688		
**Sliding Semi-landmarks**						
Ethnicity	3.07	0.0525719	3.66866E-05	1433	10.2	< .0001
Sex	1.75	0.02997836	0.00002092	1433	5.81	< .0001
Age	8.72	0.1492267	0.000026034	5732	7.24	< .0001
Individual	86.13	1.47466996	3.5982E-06	409838	2.48	< .0001
Digitizing Error	0.33	0.00564375	9.846E-07	5732		
Total	100	1.71209067	8.82234E-05	424168		
**Overall Landmarks**						
Ethnicity	3.12	0.05389094	3.60957E-05	1493	10.37	< .0001
Sex	1.72	0.02974129	1.99205E-05	1493	5.72	< .0001
Age	8.69	0.15002289	0.000025121	5972	7.22	< .0001
Individual	86.13	1.48630015	3.4808E-06	426998	2.51	< .0001
Digitizing Error	0.33	0.00575018	9.629E-07	5972		
Total	100	1.72570545	8.55809E-05	441928		

SS: sum of squares; MS: mean square; DF: degrees of freedom; F: F-value; P: P-value

### CVA/DA results

Discrimination among groups is analysed independently on the averaged ethnicity, sex, and age; both CVA (plots not shown) and DA indicate that each studied taxon is clearly distinct from one another when pooled within-group variation. Procrustes distance among ethnicity (white British vs. white Irish) is 0.0324, p < 0.0001; Procrustes distance among sex (male vs. female) is 0.02, p < 0.0001. The Procrustes distance among age is shown in Table 4 due to its matrix representation. All in 10,000 pairwise permutation tests between taxa. All permutation tests indicate that the mean shapes differed significantly among taxa.

**Table 4:** Procrustes distances matrix (lower) and P-values (upper) of age groups.

Procrustes/P-values	12 (years) Below	13–19	20–29	30–49	50 (years) Above
**12 (years) Below**		0.0003	< .0001	< .0001	< .0001
**13–19**	0.0326		0.0070	< .0001	< .0001
**20–29**	0.0522	0.0280		0.0240	0.0123
**30–49**	0.0595	0.0376	0.0166		0.0076
**50 (years) Above**	0.0637	0.0400	0.0206	0.0189	

**Fig 5:**
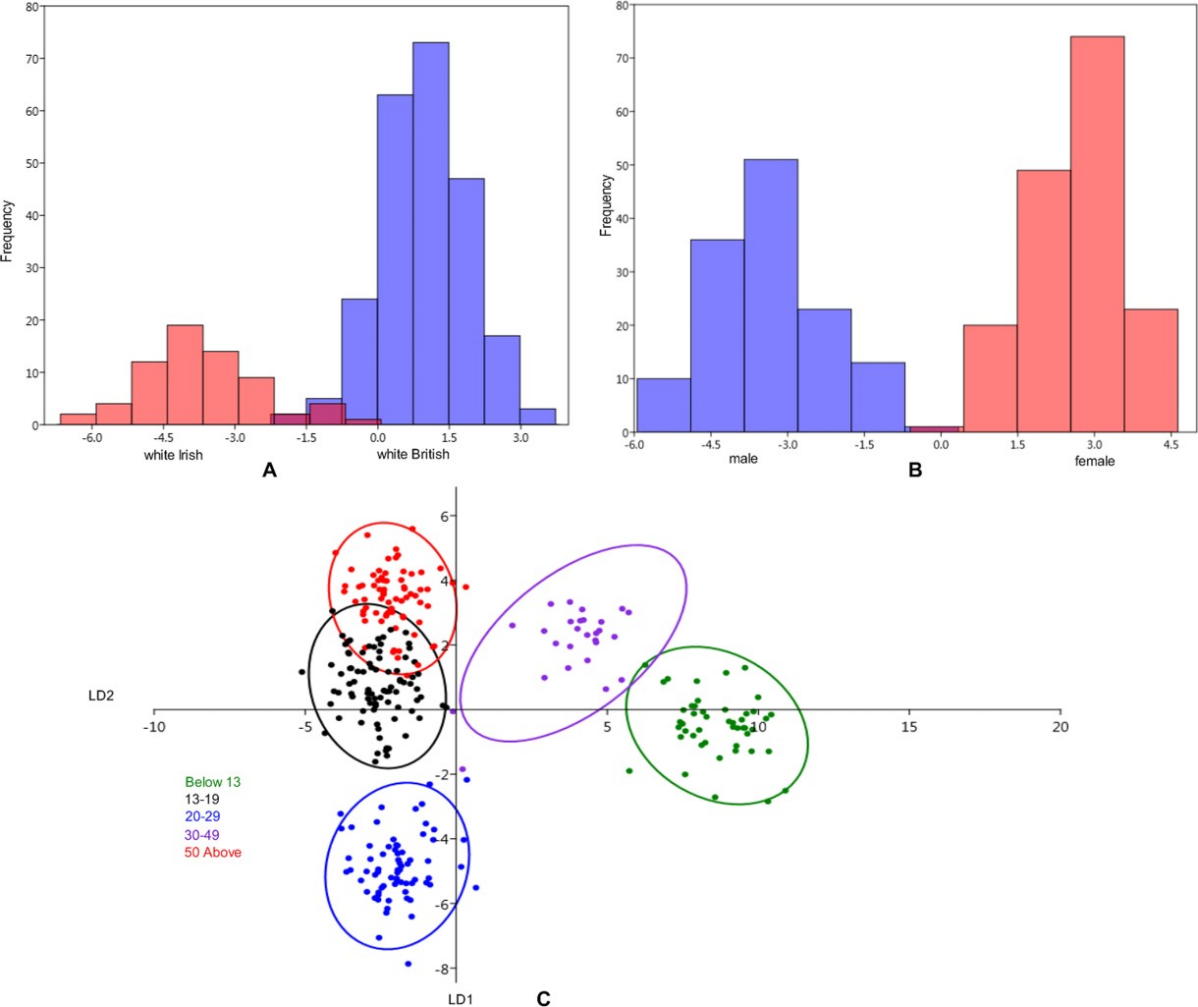
Discriminant analysis of ethnicity, sex, and age using soft-tissue crania shape after averaging. A. Between ethnicity (white British and white Irish). B. Between sex (Male and Female). C. Scatter plots of canonical variate analysis among the age classes: children (below 13 years), teenagers (13–19 years), young adults (20–29 years), adult (30–49 years), and senior adults (50 years and above); at 95% confidence ellipse.

From DA in Fig 5, the cross-validation highlights the greater differences in craniofacial shape among the groups. For the comparison between the groups, the percentage of ethnicity correctly classified is 97.67%; the percentage of sex correctly classified is 99.67%, and the percentage of age correctly classified is 98.67%. We further present the confusion matrix of each group classification in Table 5.

**Table 5:** Confusion matrix of percentage classification of ethnicity, sex, and age.

Ethnicity	White British	White Irish	Age	< 13	13–19	20–29	30–49	> 50
White British	99.14	0.85	< 13	100	0	0	0	0
White Irish	7.46	92.54	13–19	0	96.29	0	0	3.7
			20–29	0	0	98.59	1.41	0
**Sex**	**Male**	**Female**	30–49	0	0	1.16	97.67	1.16
male	99.25	0.75	50 >	0	0	0	0	100
female	0	100						

### Allometry, regression and shape variation

**Fig 6:**
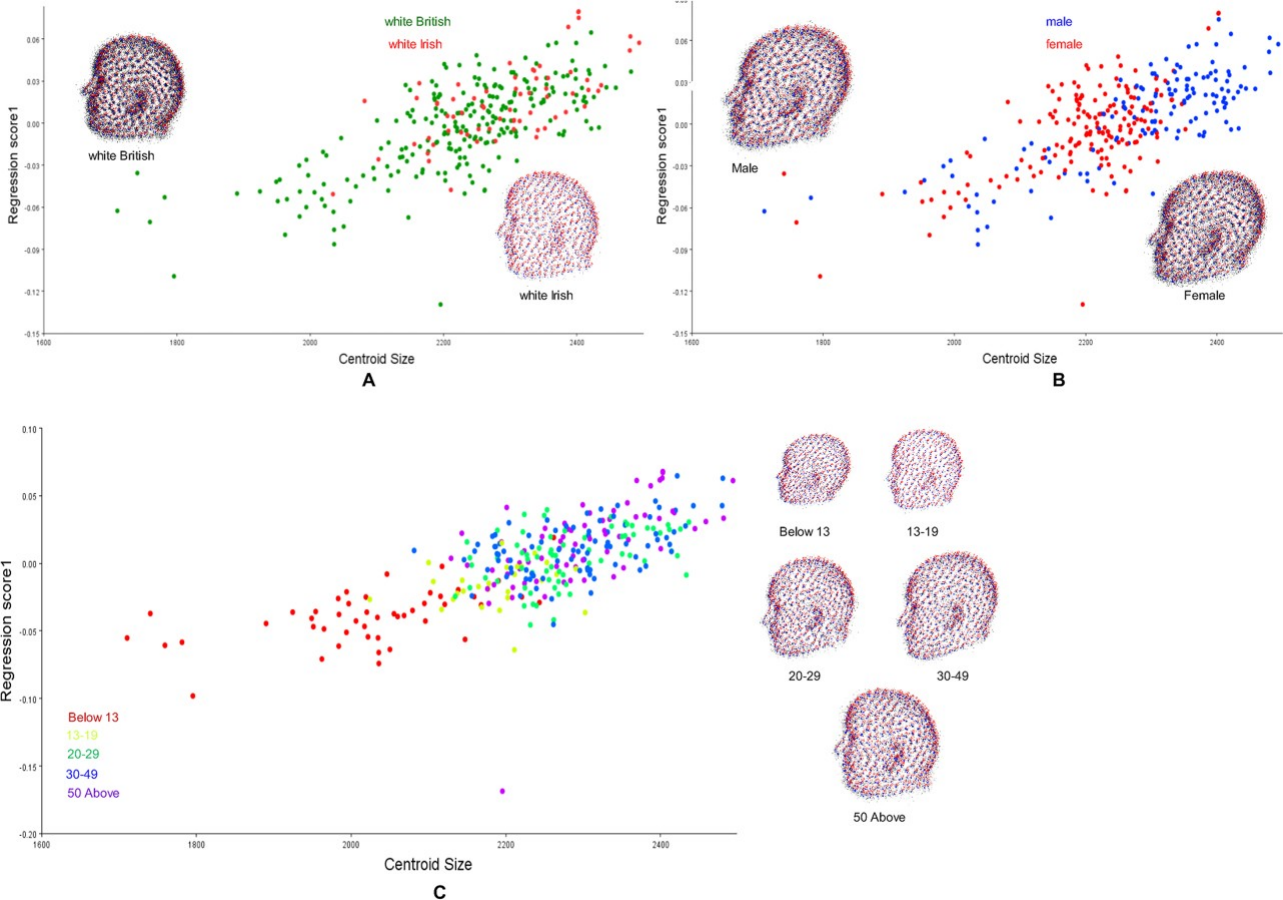
Scatter plot obtained by regression of shape onto the size. A. Between white British and white Irish. B. Between Males and Females. C. Among the Age classes: children (below 13 years), teenagers (13–19 years), young adults (20–29 years), adults (30–49 years), and senior adults (50 years and above).

All specimens are scaled to unit centroid size (CS). Rotation and translation parameters are estimated to minimize the sum of squared distances between each soft-tissue craniofacial landmark and those of an iteratively computed mean configuration. Allometry is tested for symmetric components of the averaged groups. In detail, for ethnicity, sex, and age; significant allometric patterns of symmetric variation are detected, with 9.35%, 9.95%, and 2.87%, respectively, all with p < 0.0001. The results of the linear regression of centroid size showed statistically significant patterns (Fig 6). The change in the centroid size demonstrated that simple allometry explains the changes observed in each group.

Furthermore, the total variation of each dependent variable is partitioned by the regression model into a component of variations. These components are computed for each variable separately. The residual and predicted components are expressed as a percentage of the total variation, which is intuitively useful to quantify the relative importance of allometry for the shape variation in each sub-divided dataset [37,73]. The results of the percentage predicted and significant level are shown in Table 6. All demonstrate statistically significant shape variation except the young adult age category of the age group (percentage predicted = 2.124%, p = 0.1121) which is not statistically significant and predicts the smallest percentage of shape variation.

**Table 6:** Regression results showing percentage predicted and p-values for each group.

Var	%Predicted	P-value
**Ethnicity**		
WB	9.47	< .0001
WI	7.35	< .0001
**Sex**		
Male	13.34	< .0001
Female	6.15	< .0001
**Age**		
Below 13	5.91	0.0025
13–19	7.04	0.0442
20–29	2.12	0.1121
30–49	3.25	0.0038
50 Above	9.31	< .0001

10,000 permutations test

### MANOVA results

CS and resulting shape variables are used in subsequent multivariate analyses. Using the PCA scores, multivariate analysis (MANOVA) is performed to test the significant effect of ethnicity and size on the shape of sex and age. A MANOVA is applied to the symmetric component of the ethnicity to test for differences in significant allometric trajectories of ethnicity, sex, and age with regard to two features: slopes (ethnicity × CS effect, sex x CS effect, and age x CS effect) of the regression lines and their intercepts [70].

The MANOVA results are presented in Table 7. The MANOVA procedure detects significant intercept and slope interaction on all tested variables. Size does have a significant effect on shape in ethnicity, sex, and age; for both intercept and slope. This suggests that smaller and larger individuals with the same ethnicity and sex are not similar in shape. More so, the results demonstrate that age classes are not similar in shape.

**Table 7:** MANOVA results in terms of Wilks’ Lambda.

Effect	Wiki	DF num	DF den	F	P
Ethnicity x CS	0.4159	101	199	2.767	<0.000
Ethnicity	0.4172	100	200	2.794	<0.000
Sex x CS	0.2512	101	199	5.872	<0.000
Sex	0.2514	100	200	5.955	<0.000
Age X CS	0.01354	404	786.5	3.764	<0.000
Age	0.01363	400	790.4	3.811	<0.000

## Discussions

### Procrustes ANOVA

In this study, soft-tissue craniofacial variability is investigated for two ethnicities of a subset of white Europeans. In this study, 292 soft-tissue craniofacial landmarks are analysed morphometrically and classified. The primary focus is on the analyses of shape symmetry and the allometric relationships between each ethnic group, but the differences between sex and age groups are also analysed. Using a Procrustes ANOVA [71], the significance and percentage of variability are explained. Size results show that the three effects analysed (ethnicity, sex, and age) are statistically significant (p < 0.0001); and moreover, age explains largest part of the size variability. The percentage of variability relative to the digitization error is always negligible, in the three partitions. Small measurement errors (manual landmarks = 0.0113, sliding semi-landmarks = 0.0056, and Overall = 0.0057) showed that the landmarks can be annotated with precision using the proposed method (p < 0.0001). The sliding semi-landmarks demonstrate better performance in terms of digitization over manually placed landmarks. This could be as a result of the difficulty in pinpointing the landmark locus [59]. Overall, the results of the Procrustes ANOVA indicate significant craniofacial shape differences among ethnicity, sex, and age.

### Shape variability, allometry and MANOVA

**Fig 7:**
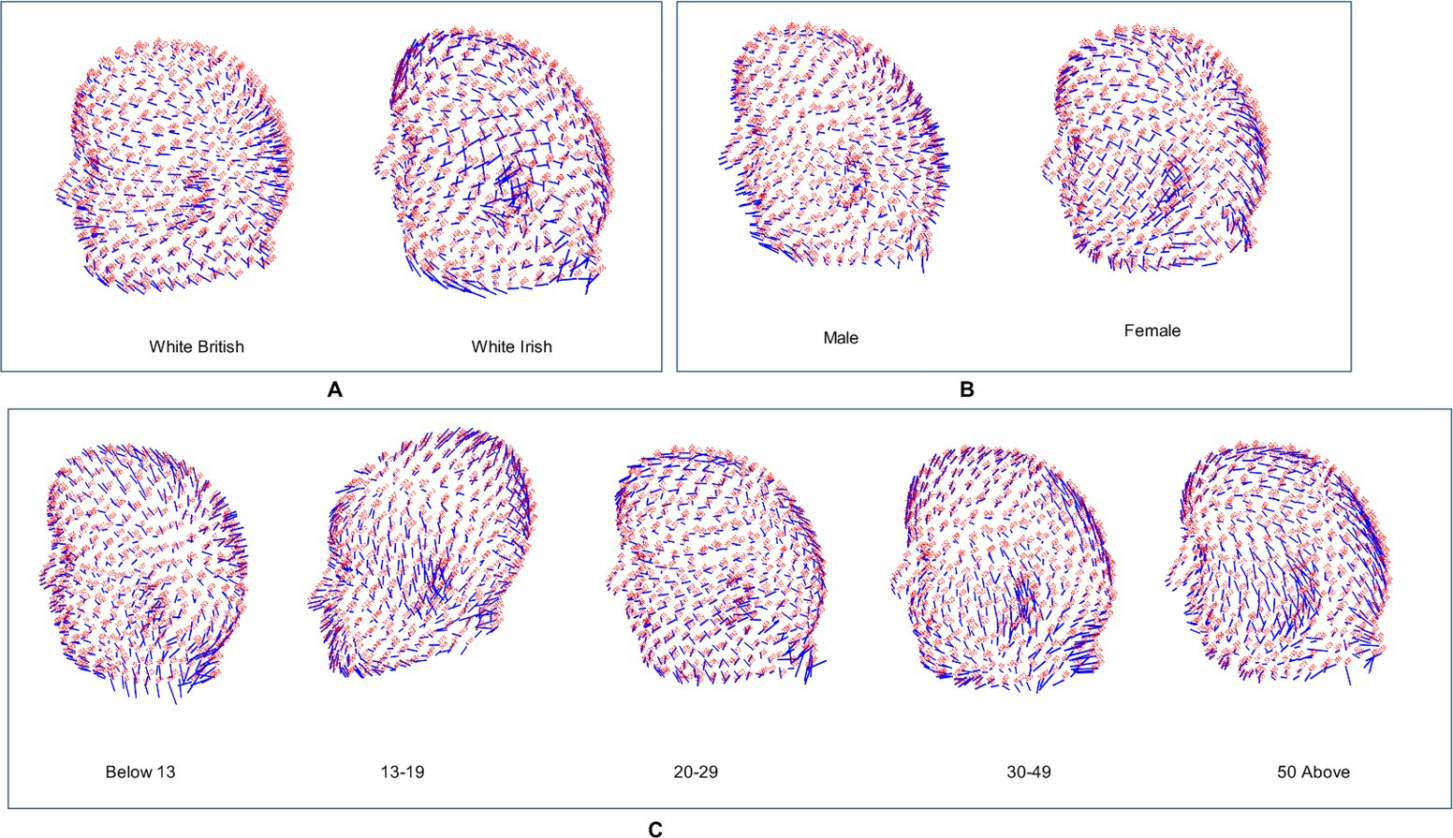
Lollipop graph of shape variability. Morphological differences between the average of each group (ethnicity, sex, and age) and average estimated 3D faces using PCA.

In order to investigate the overall variation for the entire selected sample, Principal components analysis is performed on all specimens. These analyses are carried out at different levels and the symmetric were analysed. For all computed PCs, PC1 explained almost half of the total variation, which indicates that shape variation is concentrated in a single dimension of the shape space [66]. To visualize the affected region of shape variability among ethnicity, sex and age groups, the first PC of each group is analysed more critically as presented in Fig 7. In the lollipop graphs, each of those blue circles is the average position of the landmarks we selected and the red color represents the number of each landmark. The length of the sticks tells us which way things change along the principal components [6]. Due to interpretation challenge in the lollipop graph, we further analysed the distance measurement of all selected anatomical points using EDMA and we present the results in Tables 8 and 9.

**Table 8:** Anthropometric linear distances of selected craniofacial landmarks for sex group.

	Landmark distance	Male	Female
Cranial	me-op	2.30	2.28
Face	me-n	1.74	1.73
	me-gn	2.24	2.21
	tl-tr	2.19	2.18
Eye	enl-enr	1.93	1.92
	exl-exr	1.57	1.56
Nose	n-pr	1.68	1.64
	all-alr	1.39	1.38
Mouth	chl-chr	1.67	1.63
	ls-li	1.19	1.15
Chin	li-gn	1.46	1.45
	tl-gn	2.19	2.16
	**Total**	**21.56**	**21.29**

**Table 9:** Anthropometric linear distances of selected craniofacial landmarks for age group.

		Children		Teenagers		Young adults		Adults		Senior adults	
	Distance	M	F	M	F	M	F	M	F	M	F
Cranial	me-op	2.29	2.27	2.30	2.29	2.30	2.29	2.30	2.29	3.22	3.20
Face	me-n	1.77	1.69	1.72	1.74	1.73	1.74	1.73	1.75	1.73	1.70
	me-gn	2.26	2.18	2.23	2.25	2.24	2.23	2.24	2.24	3.24	3.19
	tl-tr	2.20	2.16	2.20	2.20	2.19	2.18	2.20	2.19	2.19	2.17
Eye	enl-enr	1.94	1.90	1.94	1.93	1.93	1.93	1.93	1.94	1.93	1.90
	exl-exr	1.57	1.55	1.59	1.59	1.57	1.57	1.57	1.58	1.57	1.55
Nose	n-pr	1.69	1.58	1.69	1.69	1.68	1.66	1.68	1.68	1.69	1.60
	all-alr	1.39	1.33	1.41	1.44	1.39	1.39	1.39	1.38	1.39	1.37
Mouth	chl-chr	1.68	1.59	1.69	1.69	1.68	1.65	1.67	1.66	1.68	1.61
	ls-li	1.24	1.16	1.15	1.13	1.15	1.13	1.19	1.20	1.19	1.12
Chin/Jaw	li-gn	1.38	1.38	1.44	1.42	1.48	1.44	1.48	1.45	1.48	1.49
	tl-gn	2.11	2.09	2.16	2.14	2.21	2.17	2.21	2.17	2.22	2.17
	**Total**	**21.52**	**20.87**	**21.51**	**21.50**	**21.56**	**21.36**	**21.57**	**21.53**	**22.71**	**22.44**

Influence on shape is looked into under the three effects. Regarding the ethnicity, white British shows a narrow cranium, whereas white Irish shows a round cranium; and there is more protrusion in the British frontal face than in Irish. Though, no distance measurement was taken on ethnicity group. But regarding the sex influence on shape, a clear effect is identified in the analysis. Based on the centroid size and distances measured of the cranium and face in sex, males show a relatively larger size and sexual dimorphic in mouth width (chelion left-chelion right), mouth height (labiale superius-labiale inferius), nasal height (nasion-pronasale), and nasal bridge length (alare left-alare right), intercanthal width (endocanthion—endocanthion), biocular width (exocanthion-exocanthion), chin length (labiale inferius-gnathion), jaw height (tragion—gnathion), cranial width (metopion-Opisthocranion). It is demonstrated that nasal region increases anteriorly and posteriorly in males than in females. These results indicate that most soft-tissue features of the human head and face show strong evidence of sexual dimorphism which is in alignment with most previously published studies of craniofacial sex differences [11,18,41,74,75]. These included minimum frontal width, nasal protrusion, nasal bridge length, labial fissure width, and measures of mouth height, in white Europeans and other populations.

Regarding the age influence on shape, there is an identification of a clear effect in the analysis. The results demonstrate a slightly increase in size among the age classes. More so, the statistically significant difference among age groups is found when the entire cranium size is compared per age group and in the distances measured. The craniofacial features possess consistently larger intra-class variance due to, among others, the cranium changes relative to the increase in body size [76].

In the children group, males show a slightly larger cranial width. No difference in forehead height but males show longer facial height and wider facial width. The intercanthal width and biocular width in males are slightly longer than those of females. There is no difference in the nasal bridge but the nasal height in males is longer than females. Both mouth width and mouth height are longer in males than in females. There is no difference in the chin length but the jaw length is longer in males.

In the teenagers group, unlike in the children, there is no difference in the cranial width. But facial width, facial height, and forehead length are larger in males. There is no difference in intercanthal width but females biocular width is slightly wider. The nasal bridge length is longer in males than in females but the nasal height is longer in females than in males. Both mouth width and mouth height are larger in females than in males and both the jaw length and chin height are longer in males than in females.

In the young adults group, cranial width is longer in males. Facial width, facial height, and forehead length are larger in males than in females. The intercanthal width and biocular width are slightly wider in males than in females. The nasal bridge length and nasal height are longer in males than in females. Both mouth width and mouth height are larger in males than in females; both jaw length and chin length are longer in males than in females.

In the adults group, like in young adults group, cranial width is longer in males. Facial width, facial height, and forehead length are larger in males than in females. The intercanthal width and biocular width are slightly wider in males than in females. The nasal bridge length and nasal height are longer in males than in females. Both mouth width and mouth height are larger in males than in females; both jaw length and chin length are longer in males than in females.

In the senior adults group, the variations follow that same pattern as in adults group. Except in the mouth width where no difference is observed and also females show a longer jaw length than males. These correspond with previous studies in [19,74,77,78].

Generally, cranial width is longer in adults; facial width is longer in senior adults; facial height is longer in young adults and senior adults, and the forehead is longer in young adults. The intercanthal width is longer and equal in young adults, adults and senior adults; whereas biocular width is longer only in senior adults. Nasal bridge and nasal height are both longer in senior adults. Mouth width is longer in senior adults and mouth height is longer in young adults. Lastly, both jaw length and chin height are longer in senior adults.

It is examined in this study that allometry has a heterogeneous effect on the soft-tissue craniofacial morphology and that variation occurs in specific regions due to changes in the pattern of growth and development [79]. The results of the analysis show that the changes in craniofacial morphology over time are not associated with changes in centroid size. However, there is a statistically significant difference in craniofacial shape in both ethnicities and when traced among sex groups (p < 0.0001). Also, there is a significant difference when the size of the head shape is compared between males and females per age group (p < 0.0001). Though, the study has not examined separately the lower face and the whole cranium, which are the regions most affected by age-related morphological changes [80]. The reason why the current study did not explore these areas separately is that it aims to examine whether different age groups should be pooled. It is also clear that there is a considerable overlap in regional shape present between age groups which indicates that craniofacial aging is not simply a soft tissue phenomenon, but involves alteration in the underlying soft-tissue crania architecture [81].

Centroid size is not log-transformed, as the transformation makes no appreciable difference in the results (not shown). Regressions of shape onto size of each group are performed at a time and are statistically significant except in young adults (p = 0.1121). This is an indication of negligible allometry. Further statistical analysis is performed to be more confident in the results. In this study, we run MANOVA, which is a simple way to test the effect of size on shape when various groups are compared. Subsequently, the characteristics (i.e. slope and intercept) of the allometric trajectories of each group are tested using MANOVA, which explains significant portions of the overall variation. Regarding the allometric trajectories of ethnicity, the interaction term (test for slopes) is statistically significant (p < 0.000). When the size effect is removed (test for intercepts) and the MANOVA is repeated, the result is still statistically significant (p < 0.000). This suggests that the effect of size on shape is strong, and not similar in the two ethnicities. A significant test for intercepts means that there is support for ethnicity differences using the available samples when the effect of size on shape variation is held constant.

Regarding the allometric trajectories of sex, the interaction term is statistically significant (p < 0.000). When the size effect is removed and the MANOVA is repeated, the result is still statistically significant (p < 0.000). This suggests that the effect of size on shape is strong and not similar in the sex group. For the allometric trajectories of age class, the interaction term is statistically significant (p < 0.000). When the size effect is removed and the MANOVA is repeated, the result is still statistically significant (p < 0.000). This suggests that the effect of size on shape is strong and not similar in the age group. As it is expected since age class has larger phenotypic variations, the allometric trajectories are largely aligned with the vector of mean shape differences.

### Discriminant analysis

While the MANOVA and contrast tests detect significant shape differences in each group, DA is further employed to classify each group with moderate cross-validation rates. For ethnicity, the results show that allometric variation is negligible with respect to the ethnicity differentiation; indeed, the sample of white British are correctly classified with 99.14% and white Irish with 92.54%. For sex, 99.25% of males are correctly classified while 100% of females are correctly classified, which shows negligibility in allometric variation with respect to sex. Subsequently, the age group data followed no trend wherein the highest correct classification rates are found in children (100%) and senior adults (100%), followed by young adult (98.59%), then adults (97.67%), and finally teenagers (96.29%). The children and the senior adults age groups are found to exhibit the most variance of the five examined age groups.

The discrimination we observed in the children and adult groups is as a result of the variation in the chin, nose, forehead and crania between the age groups because of biological reasons and activity patterns. Tome et al. [82] proposed that the best facial region with the high variability power in face recognition are the forehead and nose. Though, the variability superimposition of soft-tissue is more debatable in age-related subjects than those of the hard tissue due to the changes in the surfaces [78]. Consequently, the foreheads in children proposed in [11] protrude forward and become more retro-positioned as they get older until their foreheads actually slopes posteriorly. This variation also reflects in the anterior protrusion of chin and nose as the age increases. According to Nikita [80], the cranium is one of the highly discriminative regions among age-related subjects. From our investigation, the crania size is larger in the adult age group and smaller in the children age group with more than two millimetres increase; which apparently increases the chance of discrimination between the groups.

## Conclusions

The study led to the following conclusions relating to soft-tissue craniofacial shape and size variation with ethnicity, sex, and age. Craniofacial size, expressed as centroid size, is less affected by ethnicity. But has a great impact on sex and age. Both craniofacial shape and size are significantly sexually dimorphic, which results in statistically significant differences between males and females; though not considered in different age groups. Attention should, therefore, be given to over-classification problems when DA is applied on the craniofacial shape, when captured by multiple landmarks. However, due to the uncertainty of biological reality reflection, the assigned sliding semi-landmarks may not adequately reflect the shape of the entire head and face under study. This may also have a negative impact on biological variability within the sample related to ethnicity, sex, or age. Furthermore, we face some discrepancy challenges in the dataset used. This is because the dataset comes from various ethnicities and countries with white British having the highest number of sample, followed by white Irish. Other ethnicities are insignificant for analysis consideration due to their infinitesimal sample size. More so, no subject is found in the white Irish males of teenagers (13–19 years) age group. These discrepancies consequently have effect on the interpretation of our results, especially the principal component analysis. While further study is recommended for clarification on the aforementioned issues, this study, nonetheless, combines pragmatic solutions to configure an optimized pipeline for high-throughput multi-point craniofacial signature in 3D to the investigation of ethnicity, sex, and age related variation in craniofacial morphology. Thus, this study is limited to white Europeans descents (British and Irish), therefore the generalizability of these results to other populations cannot be assumed.

## Supporting information

S1 Data (XLSX)

S2 Data (XLSX)

S3 Data (XLSX)

S1 File (PDF)
