# Supplementary material for: Morphometric approach to 3D soft-tissue craniofacial analysis and classification of ethnicity, sex, and age
Source: PLoS One. 2020 Apr 9;15(4):e0228402. doi: 10.1371/journal.pone.0228402 (PMC7145022; doi:10.1371/journal.pone.0228402)
Supplement: S1 File — (PDF) [file pone.0228402.s004.pdf]

Use of Subject

Inbox x

Olalekan Agbolade <agbolade.ola@ieee.org>

to nick.pears

Mar 12, 2020, 10:33 AM (23 hours ago)

Dear Dr. Nick,

Agbolade Olalekan is my name from Universiti Putra Malaysia. Regarding the Headspace dataset we requested previously (license agreement attached), PlosOne Journal requested permission for the use of subject which reads thus:

As per the PLOS ONE policy (http://journals.plos.org/plosone/s/submission-guidelines#loc-human-subjects-research) on papers that include potentially identifying information, the individuals must be informed of the terms of the PLOS open-access (CC-BY) license and provide specific permission for publication of these details under the terms of this license. We would like to request clarification on whether you have obtained all relevant permissions and consent for publication of the images from the Headspace dataset.

In addition, we also require clarification on whether you have permission from the owners of the Headspace dataset to publish these images under the CC-BY license under which PLOS ONE articles publish (https://journals.plos.org/plosone/s/licenses-and-copyright). We would like to ask you to provide us with proof that the owner of that content (a) has given you written permission to use it, and (b) has approved of the CC-BY license being applied to their content.

We want to know if we are free to use the subjects for research publications under PLOS open-access (CC-BY) license and if there is any written permission from individual participant to use their subjects. Some figures are also attached which are used in the journal to describe methodology.

Thank you so much and kind regards

3 Attachments

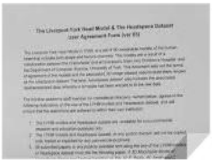

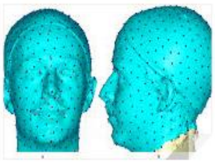

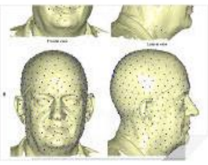

Nick Pears

to me

Mar 12, 2020, 7:12 PM (15 hours ago)

Dear Olalekan

Yes - no problem - you can publish anything that we gave you. The subjects signed a form with consent to publish.

Nick

•••

--

Nick Pears

http://www-users.cs.york.ac.uk/nep/

Department of Computer Science

University of York

Disclaimer

Thank you very much. | Many thanks. | Thank you for the clarification.
